# Immune modulating effects of cyclophosphamide and treatment with tumor lysate/CpG synergize to eliminate murine neuroblastoma

**DOI:** 10.1186/s40425-015-0071-3

**Published:** 2015-06-16

**Authors:** Jill A. Gershan, Kristen M. Barr, James J. Weber, Weiqing Jing, Bryon D. Johnson

**Affiliations:** Division of Hematology/Oncology/Transplant, Department of Pediatrics, Medical College of Wisconsin, Milwaukee, WI 53226 USA; Carroll University, Waukesha, WI 53186 USA

**Keywords:** Neuroblastoma, CpG, Lymphodepletion

## Abstract

**Background:**

Neuroblastoma is a pediatric cancer of neural crest origin. Despite aggressive treatment, mortality remains at 40 % for patients with high-risk disseminated disease, underscoring the need to test new combinations of therapies. In murine tumor models, our laboratory previously showed that T cell-mediated anti-tumor immune responses improve in the context of lymphopenia. The goal of this study was to incorporate lymphodepletion into an effective immune therapy that can be easily translated into neuroblastoma standard of care. Based on the lymphodepleting effects of cyclophosphamide, we hypothesized that cyclophosphamide would synergize with the TLR9 agonist, CpG oligodeoxynucleotide (ODN), to produce a T cell-mediated anti-neuroblastoma effect.

**Methods:**

To test this hypothesis, we used the AgN2a aggressive murine model of neuroblastoma. Mice bearing subcutaneous tumors were treated with cyclophosphamide followed by treatment with tumor cell lysate mixed with CpG ODN injected at the tumor site.

**Results:**

Subcutaneous neuroblastoma regressed only in mice that were treated with 100 mg/kg cyclophosphamide prior to receiving treatments of tumor lysate mixed with CpG ODN. The anti-neuroblastoma response was T cell-mediated. Synergy between cyclophosphamide and the tumor lysate/CpG ODN treatment influenced the production of anti-tumor CD8 T cell effectors, and dendritic cell homeostasis. For clinical consideration, an allogeneic tumor lysate was used effectively with this protocol to eliminate AgN2a tumor *in vivo*.

**Conclusion:**

Synergistic immune modulating effects of cyclophosphamide and a treatment containing tumor cell lysate and CpG ODN provide T cell-mediated anti-tumor activity against murine neuroblastoma.

**Electronic supplementary material:**

The online version of this article (doi:10.1186/s40425-015-0071-3) contains supplementary material, which is available to authorized users.

## Background

Neuroblastoma is a pediatric cancer of neural crest origin that accounts for 12 % of all pediatric cancer deaths. Patients with localized neuroblastoma in the adrenal medulla or paraspinal ganglia respond well to conventional therapy with an overall survival of greater than 95 %. Unfortunately, nearly half of all neuroblastoma patients have high-risk disseminated disease for which overall survival is poor [1, 2]. Treatment with myeloablative chemotherapy followed by autologous stem cell transplant and isotretinoin has improved survival, but there is still greater than 60 % mortality. Based on a 2-year 20 % increase in event-free survival, anti-GD2 disialoganglioside antibody in combination with interleukin-2 (IL-2) and granulocyte colony stimulating factor (GM-CSF) has been added to standard of care protocols [3]. This increase in survival is proof of principle that immune therapy can be effectively administered for treatment of refractory disease. However, there is a need to test other therapies to further improve survival.

The goal of immune therapy is to destroy tumors through the activation of immune cells, including T lymphocytes. In a murine model of aggressive neuroblastoma (AgN2a), we previously observed a T cell-mediated immune response resulting in elimination of established AgN2a [4]. In this model, mice were administered a cell-based tumor vaccine expressing immune co-stimulatory molecules, CD54, CD80, CD86 and CD137L. Importantly, this cell-based tumor vaccine failed to eliminate established tumor unless it was administered in the context of lymphodepletion. The best anti-neuroblastoma response occurred when the vaccine was delivered following hematopoietic stem cell transplantation (HSCT) with an adoptive transfer of tumor antigen sensitized lymphocytes depleted of CD25^+^ cells [5].

In a multiple myeloma model, we observed a T cell-mediated anti-tumor response when mice were given checkpoint blockade in the context of lymphodepletion [6]. Notably, lymphodepletion followed by anti-PD-L1 or anti-PD-1 administration was ineffective at producing a T cell mediated anti-tumor response for a PD-L1-negative murine neuroblastoma. These data emphasize that anti-tumor immune therapy must be tailored specifically toward tumor characteristics in order to be effective. Yet, these previous studies demonstrated that lymphocyte activation in the context of lymphodepletion appears to be essential to enhance anti-tumor immunity.

For the current study, our aim was to test the anti-tumor efficacy of CpG oligodeoxynucleotide (ODN) 1826, a class B toll-like receptor 9 (TLR9) agonist, (henceforth referred to as CpG) in the context of non-myelablative chemotherapy. Cyclophosphamide (Cy) was delivered as a lymphodepleting agent prior to administration of a tumor cell lysate plus CpG (lysate/CpG) treatment. Tumor lysate was combined with CpG in an effort to augment antigen capture by antigen presenting cells. The agents used in this study are clinically translatable and can be tested for synergy with existing standard of care and other immune modulating therapies for the treatment of neuroblastoma.

Innate immune cells integrate a variety of signals from the microenvironment that allow them to mature and promote an adaptive anti-tumor immune response. Since tumor microenvironments are generally immunosuppressive, the maturation and/or activation of innate immune cells can be suppressed, contributing to immune tolerance to tumor. TLR9 ligands are synthetic unmethylated bacterial CpG motifs that have been under study as adjuvants to activate immune cells. Engagement of TLR9 ligands with the intracellular endosomal TLR9 receptor mimics the “danger signal” that is activated by bacterial DNA. This results in activation and maturation of innate immune cells such as natural killer (NK) cells [7], macrophages [8] and dendritic cells (DCs) [9]. Upon maturation of innate cells there is an up-regulation of cellular mechanisms that promote a Th1 adaptive immune response, including efficient antigen processing and presentation [10].

Cyclophosphamide also has immune modulating effects. Studies have shown that Cy induces Th1 cytokine production and differentiation of Th17 cells [11, 12], transiently reduces T regulatory cell numbers [13], induces type I interferon [14], and enhances DC activation and DC-T cell priming [15–18]. The lymphopenia induced by Cy is transient and is associated with *in vivo* proliferation and expansion of antigen-specific T cells [19].

CpG has been shown to synergize with the immune modulating effects of Cy to enhance antitumor immunity. The administration of the human-specific class B CpG ODN 2006, in combination with Cy improved survival in mice with rhabdosarcoma [20]. In this model, the enhanced anti-tumor response was T cell dependent. In addition, administration of CpG ODN 1826 with agonist anti-CD40 antibody in combination with vincristine, Cy and doxorubicin chemotherapy produced anti-tumor effects mediated by effector M1 macrophages [21]. Based on the known immune modulating mechanisms of Cy and CpG, we hypothesized that Cy would synergize with lysate/CpG treatment to produce a T cell-mediated anti-neuroblastoma response. The results demonstrate that administration of Cy prior to lysate/CpG treatment is necessary to promote a T cell-mediated regression of established neuroblastoma.

## Results

### Lysate/CpG provides protection from live tumor challenge and delays progression of established tumor

**Fig. 1:**
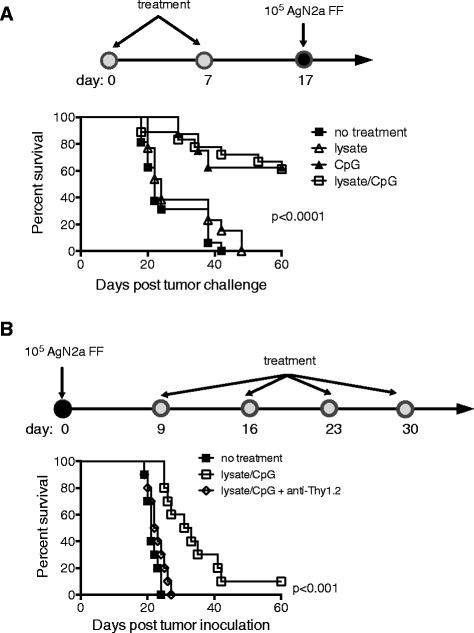
Lysate/CpG treatment protects from live tumor challenge and delays growth of existing neuroblastoma. **a** Groups of mice (6–8 per group) received 2 weekly hind flank sc treatment of AgN2a tumor lysate, CpG, AgN2a lysate mixed with CpG or no treatment. Mice were challenged with 10^5^ AgN2a FF cells 10 days after the last treatment and were followed for tumor growth. Mice were euthanized when tumor growth exceeded 250 mm^2^. The graph represents 1 of 2 separate experiments. The CpG only group was tested once with 8 mice per group. **b** Mice received 10^5^ AgN2a FF cells sc in the hind flank. Beginning 9 days after tumor inoculation, mice received no treatment or 4 weekly treatments of lysate/CpG. In addition to lysate/CpG, some mice received 200 μg of anti-Thy1.2 antibody ip on days 14, 18, 22 and 26. Mice were followed for survival and euthanized when tumor size reached 250 mm^2^. The graph represents combined data from 2 experiments with 5 mice per group. Data was analyzed for statistical significance using the log-rank (Mantel-Cox) test

To begin, a tumor challenge model was used to rapidly assess the relevance of using tumor lysate and CpG for the treatment of neuroblastoma. Mice received 2 weekly hind flank subcutaneous (sc) treatments of tumor lysate, CpG or a combination of lysate/CpG (Fig. 1a). Ten days later mice were challenged with 10^5^ live AgN2a cells expressing firefly luciferase (AgN2a FF) at the treatment site. Mice were followed for tumor growth and euthanized when growth exceeded 250 mm^2^. All of the mice that received only lysate died from tumor burden by 48 days following tumor inoculation (Fig. 1a). Of the mice that received CpG only or lysate/CpG, 5 of 8 and 11 of 18, respectively, survived for 60 days without any palpable tumor mass at the inoculation site. These data show that a treatment containing CpG or lysate/CpG induced an immune response to AgN2a tumor cells capable of preventing tumor formation in some tumor challenged mice.

It has been reported that DCs do not spontaneously cross-present necrotic cell antigen to CD8 T cells [22]. Therefore, in order to optimize antigen capture by antigen presenting cells, lysate/CpG versus CpG alone was used as treatment in a therapeutic model. To test if the lysate/CpG treatment would eliminate existing tumor, mice were first inoculated sc with 10^5^ AgN2a FF cells. Nine days after tumor inoculation mice received either no treatment or 4 weekly peri-tumoral sc treatments of lysate/CpG (Fig. 1b). In addition to lysate/CpG, some mice were given intraperitoneal (ip) injections of the T cell depleting anti-Thy1.2 antibody (200 μg). The anti-Thy1.2 antibody was administered on days 14, 18, 22 and 26 according to the schedule in Fig. 1b. T cell depletion was confirmed in all anti-Thy1.2 treated mice by flow cytometric analysis of CD4 and CD8 T cells in peripheral blood on day 21 (data not shown). All of the mice that received no treatment or lysate/CpG with anti-Thy1.2 were euthanized for tumor burden by 27 days after tumor inoculation (Fig. 1b). Of the mice that received the lysate/CpG treatment, tumor growth was significantly delayed, and 1 of 10 mice had no palpable tumor mass 50 days after tumor inoculation. Since the anti-tumor effect was abrogated when mice received the anti-Thy1.2 antibody, the delay in tumor progression appeared to be T cell-mediated.

### Cy and lysate/CpG induces IFN-γ producing tumor-specific CD8 T cells

**Fig. 2:**
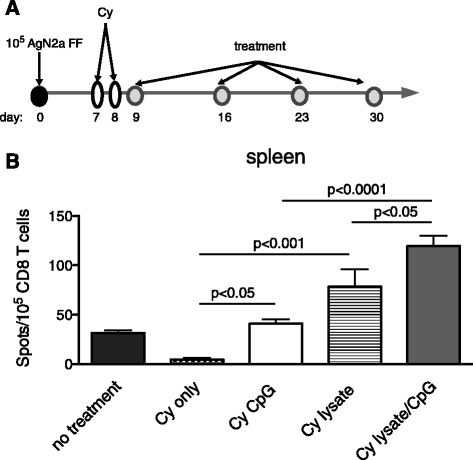
CD8 T cell IFN-γ is increased in mice that receive Cy and lysate/CpG. **a** Groups of mice were treated (no treatment, Cy only, Cy and CpG, Cy and lysate or Cy and lysate/CpG) according to the schedule. On day 22, spleens from 3 mice per group were harvested and pooled. **b** CD8 T cells were purified by immunomagnetic cell separation and 10^5^ were incubated overnight with 10^5^ AgN2a stimulator cells. IFN-γ production was analyzed per ELISPOT assay. Background signal (T cells only with no tumor cell stimulators) was subtracted from the experimental values. The graph represents data from 1 to 2 independent experiments. Data was analyzed for statistical significance using one-way ANOVA

Cy is a widely used standard of care chemotherapeutic that has immune modulating effects [23]. Low dose Cy is know to transiently deplete T regulatory (T regs) cells [13], as well as induce a lymphopenia that contributes to homeostatic proliferation of lymphocytes. Since our previous data has shown that anti-tumor immunity is enhanced in the context of lymphopenia, Cy was administered prior to immune modulating treatment according to the schedule in Fig. 2a. To determine which immune modulating agent(s) were most effective in producing anti-tumor effector T cells, mice were treated with Cy only, Cy and CpG, Cy and lysate or Cy and lysate/CpG. On day 22, spleens were harvested and pooled according to treatment groups. CD8 T cells were incubated with AgN2a target cells and analyzed for IFN-γ secretion in ELISPOT assays. There was a significant increase in IFN-γ producing tumor-specific splenic CD8 T cells when mice were treated with Cy and lysate/CpG > Cy and lysate > Cy and CpG > Cy only (Fig. 2b). These data suggest that administration of Cy synergizes with lysate/CpG to produce anti-AgN2a T cell effectors. Since the Cy and lysate/CpG treatment provided the best CD8 T cell effector response, this treatment group was further tested for *in vivo* efficacy.

### Existing neuroblastoma regresses when cyclophosphamide is administration prior to lysate/CpG treatment

**Fig. 3:**
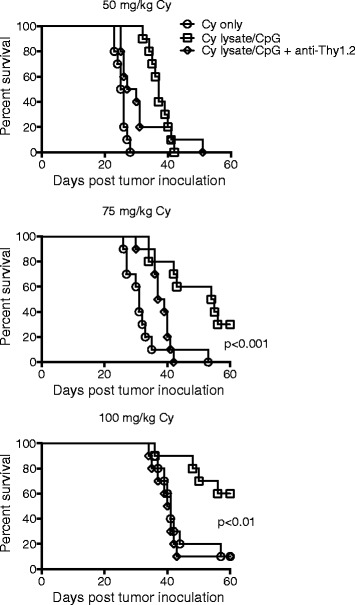
Cy administered prior to lysate/CpG treatment eliminates existing neuroblastoma. Groups of mice received ip Cy in doses of 50, 75 or 100 mg/kg followed by either no treatment or 4 weekly treatments of lysate/CpG according to the schedule in Fig. 2a. In addition to lysate/CpG, some mice received 200 μg of anti-Thy1.2 antibody ip on days 14, 18, 22 and 26. Mice were followed for survival and euthanized when tumor size reached 250 mm^2^. Each graph represents data combined from 2 separate experiments with 5 mice per group. Data was analyzed for statistical significance using the log-rank (Mantel-Cox) test

To test if the Cy and lysate/CpG treatment was therapeutic in reducing or regressing existing neuroblastoma, mice were inoculated sc with 10^5^ AgN2a FF to generate a tumor burden and were treated according to the schedule in Fig. 2a. For this experiment, different doses of Cy were used to determine if dose had any effect on anti-tumor synergy with lysate/CpG. Cy was administered at doses of 50, 75 or 100 mg/kg on days 7 and 8 after tumor inoculation. One day after the second Cy treatment, some mice began 4 weekly peri-tumoral sc treatments of lysate/CpG and all mice were followed for tumor growth (Fig. 3). For the mice that received 50 mg/kg Cy, and lysate/CpG there was a delay in tumor growth as compared to Cy only treated mice; however, all of the mice eventually died from tumor burden. For mice treated with 75 mg/kg Cy and lysate/CpG, tumor was eliminated in 3 of 10 treated mice. Mice that received 75 mg/kg Cy and lysate/CpG plus anti-Thy1.2 all died from tumor burden. These data suggest that the anti-tumor immune response is T cell mediated. The best anti-tumor effect occurred when mice were treated with 100 mg/kg Cy and lysate/CpG. With this treatment, 6 of 10 mice experienced tumor regression with no palpable tumor. In some mice tumor regression was confirmed by biophotonic imaging showing loss of tumor luciferase activity (Additional file 1: Figure S1). For the mice treated with Cy only there was a progressive delay in tumor burden when the dose was increased from 50 to 100 mg/kg. These data suggest that Cy alone has dose-dependent anti-tumor effects (Additional file 2: Figure S2). Together, these data suggest that there is anti-tumor synergy when Cy is administered prior to lysate/CpG, and the synergy appears to depend on the Cy dose. Since Cy at 100 mg/kg offered the best anti-tumor effect when combined with lysate/CpG treatment, 100 mg/kg was used in subsequent experiments.

### Lysate/CpG enhances T_EM_ expansion and activation and differentiation of DCs during Cy-induced homeostatic expansion

**Fig. 4:**
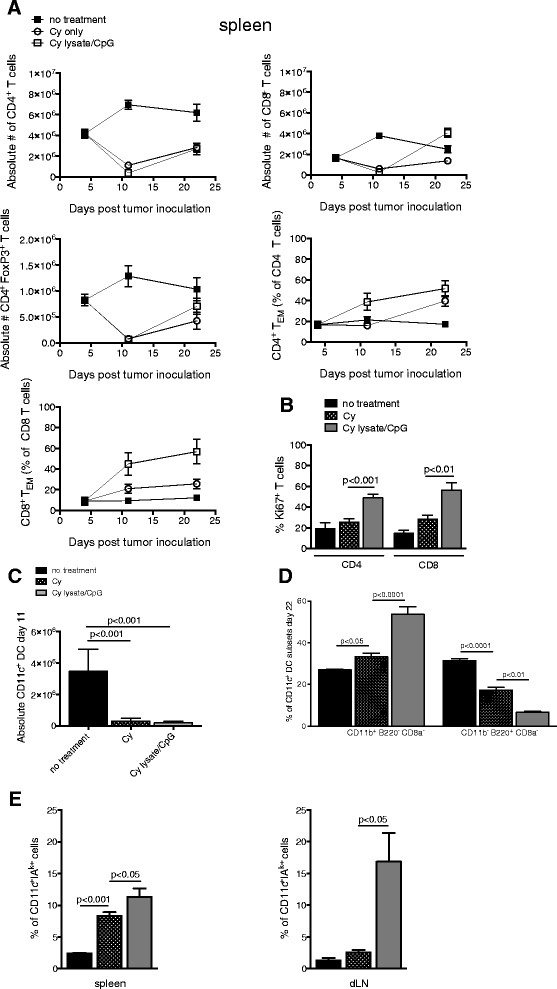
Immune cell analysis. Mice were divided into treatment groups (no treatment, Cy only or Cy and lysate/CpG) and treated according to the schedule in Fig. 2a. **a** Spleens were harvested and pooled from 5 mice in each treatment group on days 4, 11 and 22 of the treatment schedule. The depletion and recovery of CD4^+^ T cells, CD8^+^ T cells, CD4^+^FoxP3^+^ T cells, CD4^+^ CD44^+^ CD62L^−^ effector memory (EM) and CD8^+^ CD44^+^ CD62L^−^ EM cells is shown over time. **b** On day 22, spleens were harvested and pooled from 4 to 5 mice in each treatment group. CD3^+^ CD4^+^ or CD3^+^ CD8^+^ cells were analyzed for intracellular Ki67 expression. **c–e** Spleens were pooled from 3 to 5 mice in each treatment group. **c** Absolute number of CD3^−^ CD11c^+^ DCs on day 11 of the treatment schedule in Fig. 2a. **d** The percentage of spleen CD3^−^ CD11c^+^ dendritic cells differentially expressing CD11b, B220 and CD8α on day 22. **e** The percentage of CD3^−^CD11c^+^ DC expressing MHC class II (IA^k+^) on day 22. Graphs A-E represent data combined from 2 independent experiments. Data was analyzed for statistical significance using one-way ANOVA

Cy administered prior to lysate/CpG treatment was required to produce an anti-neuroblastoma response. Since Cy produces a transient lymphopenia, we sought to characterize T cell and DC subsets during homeostatic proliferation following Cy administration. We were specifically interested in determining if the anti-neuroblastoma effects of lysate/CpG treatment correlated with expansion of T cells and dendritic cell subsets that promote both adaptive and innate immune responses. To determine the effects of treatment on T cell depletion and reconstitution, splenic T cells were analyzed before Cy and several time points following Cy administration. As expected, numbers of CD4^+^ T cells, CD8^+^ T cells and CD4^+^FoxP3^+^ T regulatory cells were reduced 3 days after Cy administration (day 11 post tumor inoculation) (Fig. 4a). At 14 days after Cy administration (day 22 after tumor inoculation), there was greater expansion of CD8^+^ T cells and CD4^+^ T regulatory cells in mice treated with Cy and lysate/CpG as compared to Cy only. Using the average absolute number of splenic CD8^+^ T cells and CD4^+^ FoxP3^+^ T regulatory cells (calculated from 4 to 14 mice), the CD8^+^:CD4^+^FoxP3^+^ T cell ratio was 3.3 for mice treated with Cy and 5.7 for mice treated with Cy and lysate/CpG. The increased ratio of CD8 T cells to T regulatory cells in mice treated with Cy and lysate/CpG correlated with an improved anti-tumor response as compared to mice treated with Cy only. Of interest, the percentage of CD4^+^ and CD8^+^ T effector memory (T_EM_; CD44^+^CD62L^−^) cells was greater in mice treated with Cy and lysate/CpG > Cy only > no treatment. By 14 days after Cy administration, there was also a significant increase in the percentage of Ki67^+^ splenic CD4^+^ and CD8^+^ T cells in mice that received Cy and lysate/CpG as compared to Cy only (Fig. 4b). Together these data suggest that lysate/CpG enhances proliferation of CD4 and CD8 T cells and augments the expansion of CD8 effector T cells during Cy-induced homeostatic proliferation.

CpG has been reported to enhance DC differentiation and the capacity of DCs to activate T cells [9, 24, 25]. To determine the effect of lysate/CpG on DC differentiation during Cy-induced homeostatic expansion, CD3^−^CD11c^+^ DCs were analyzed for expression of CD11b, CD8α, B220 and the activation marker, MHC class II. The absolute number of splenic DCs was reduced 3 days (day 11 after tumor inoculation) following Cy administration (Fig. 4c). The recovery of DC subsets was analyzed 14 days following Cy administration (day 22 after tumor inoculation). In the spleen, there was a significant increase in percentage of migratory CD11b^-^B220^+^CD8α^−^ and a decrease in immature CD11b^+^B220^+^CD8a^−^ DCs when mice received lysate/CpG during the Cy-induced homeostatic expansion (Fig. 4d). During Cy-induced homeostatic proliferation, the percentage of CD11c^+^ MHC class II^+^ (IA^K^) DCs in the spleen and draining lymph node was increased in mice that received Cy and lysate/CpG (Fig. 4e). Together, these data indicate that during Cy-induced homeostatic proliferation, lysate/CpG augments DC activation and may skew DC differentiation.

### Treatment with Cy and an allogeneic lysate/CpG regresses AgN2a tumor

**Fig. 5:**
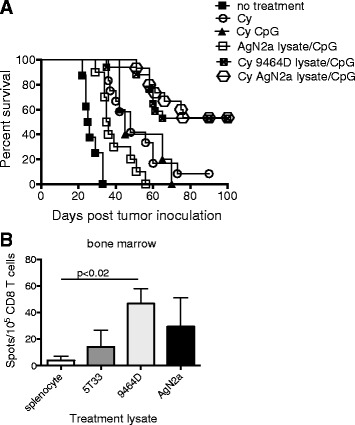
Cy and allogeneic lysate/CpG treatment is effective in regressing AgN2a tumor. **a** Groups of mice were treated with either Cy and AgN2a lysate/CpG or Cy and 9464D allogeneic lysate/CpG according to the protocol in Fig. 2a. Mice were followed for tumor growth and were euthanized when tumor reached 250 mm^2^. Nine of 17 AgN2a lysate/CpG and 8 of 15 9464D lysate/CpG treated mice did not have palpable tumor 100 days post tumor inoculation. Data was analyzed for statistical significance using the log-rank (Mantel-Cox) test. The graph represents data combined from 2 separate experiments. The Cy and CpG control group was tested once with 5 mice per group showing statistical significance. **b** Groups of AgN2a-bearing mice were treated with CpG and (1) irrelevant splenocyte lysate, (2) control 5T33 multiple myeloma tumor lysate, (3) allogeneic 9464D neuroblastoma tumor lysate, or (4) syngeneic AgN2a neuroblastoma tumor cell lysate according to the schedule in Fig. 2a. On day 22 after tumor inoculation, bone marrow was harvested and pooled from 3 mice in each group. CD8 T cells were purified by immunomagnetic cell separation and 10^5^ were incubated overnight with 10^5^ AgN2a stimulator cells. IFN-γ production was analyzed per ELISPOT assay. Background signal (T cells only with no tumor cell stimulators) was subtracted from the experimental values. The graph represents data from 1 to 2 independent experiments. Data was analyzed for statistical significance using the Student’s *t* test

To produce a more clinically translatable treatment protocol, anti-AgN2a efficacy was tested using an allogeneic lysate. Mice were treated with Cy, and an allogeneic 9464D neuroblastoma lysate/CpG or Cy and AgN2a lysate/CpG according to the schedule in Fig. 2a. Mice were followed for tumor growth and euthanized when tumors reached 250 mm^2^. Similar to previous results, 8 of 15 of the mice that received Cy plus AgN2a lysate/CpG were tumor-free 100 days following tumor cell inoculation (Fig. 5a). Importantly, 9 of 17 mice treated with Cy plus the 9464D allogeneic lysate were also tumor free. All of the control mice (untreated, treated with Cy, Cy and CpG or AgN2a lysate/CpG only) died. One control mouse that received Cy only did not develop tumor. Tumor-bearing mice treated with CpG and the neuroblastoma 9464D lysate generated AgN2a-specific T cells as shown in the IFN-γ ELISPOT in (Fig. 5b). For the ELISPOT, AgN2a cells were used as stimulator cells.

## Discussion

Our previous studies showed that the anti-tumor efficacy provided by immune-based therapies increases when the therapy is administered in the context of lymphopenia [4–6, 26, 27]. In this study, we found that the T cell-mediated anti-tumor efficacy of a tumor lysate/CpG treatment was dependent on pre-treatment with lymphodepleting cyclophosphamide chemotherapy (Fig. 3). The synergistic effects of Cy and lysate/CpG were required to induce tumor elimination as Cy alone or lysate/CpG alone was ineffective at eliminating tumor. Cy produced a transient lymphodepletion of T cells that reached a nadir within 2 days of administration (Fig. 4a). There was a gradual increase in splenic T cell absolute numbers by 14 days after Cy administration. The T cells expanding under Cy-induced lymphopenic conditions expressed T_EM_ activation markers and this was enhanced in mice that received both Cy and lysate/CpG. Others have reported an expansion of T effectors secondary to immunization with peptide and CpG [28]. These data suggest that homeostatic expansion of anti-tumor T cells is induced by lymphopenia and further augmented by lysate/CpG.

Anti-tumor T cell memory was tested in mice that had regressed AgN2a when treated with Cy and lysate/CpG. When mice were re-challenged with 10^5^ live AgN2a, there was a delay in tumor progression as compared to untreated mice. However, mice eventually died of tumor progression, indicating a lack of effective anti-tumor memory (data not shown). In a previous study, a T cell-mediated anti-rhabdomyosarcoma effect occurred when mice were treated with Cy and the human 2006 class B CpG; T cell memory was also not observed in this model [20]. The mechanisms responsible for T cell memory are unknown, but the combination of lymphodepletion with high dose tumor antigen and CpG may be a combination that drives T cell differentiation toward a terminally differentiated effector phenotype and less toward a memory phenotype [29]. Our data and the data of others indicate that despite the T cell dependent anti-tumor response induced by Cy and lysate/CpG, additional immune modulation is required to generate anti-tumor T cell memory.

Synergy of Cy with tumor vaccines/treatments to produce a T cell dependent anti-tumor effect has been previously reported [30]. Data in the current study provides insight into the contributions of Cy and lysate/CpG towards producing an anti-tumor response. Both Cy and CpG can modulate innate immunity. Reports of Cy-induced immune modulation include lymphopenia-induced recovery of immature DCs [14, 17, 30], alteration of DC subsets in secondary lymphoid organs [15, 17] and transient depletion of T regulatory cells [13]. Cy has also been shown to mobilize bone marrow DCs and promote expansion of immature DCs in peripheral blood [17, 19, 30]. It has been proposed that lymphopenia-induced myelopoiesis resets myeloid and DC homeostasis. In the current study, Cy induced a depletion of DCs and changed the balance of DC subsets in the spleen. There was a significant increase in splenic CD11b^+^ B220^−^ CD8α^−^ migratory DCs, and lysate/CpG synergized with Cy by further increasing the percentage of splenic CD11b^+^ B220^−^ CD8α^−^ migratory DCs. There was also a significant decrease in immature non-migratory CD11b^−^ B220^+^ CD8a^−^ DCs in mice treated with Cy as compared to untreated mice (Fig. 4d). Expression of MHC class II on CD11c^+^ DCs is a marker of maturation and is required for efficient antigen presentation. Cy alone induced a significant increase in the percentage of splenic CD11c^+^ DCs expressing MHC class II (Fig. 4e). Lysate/CpG synergized with Cy to further increase the percentage of CD11c^+^ spleen cells expressing MHC class II. In the dLN there was a significant increase in the numbers of cells expressing MHC class II only in mice that received Cy and lysate/CpG. These data suggest that the T cell-mediated anti-neuroblastoma response may be influenced by the synergy of Cy with lysate/CpG to reset DC homeostasis and maturation.

The biologic activities of Cy are dose dependent [31]. At 200 mg/kg, bone marrow dendritic cell precursors are depleted [30]. In this study, the best anti-tumor synergy occurred when 100 mg/kg of Cy was given prior to lysate/CpG (Fig. 3). When mice were treated with Cy only, tumor progression was delayed as the dose increased (Additional file 2: Figure S2). In fact, at 100 mg/kg, 1 of 10 mice did not form a tumor. These data indicate that Cy may have some direct cytolytic effect on the tumor cells. The induction of an efficient immune response requires both antigen access and DC maturation [22]. It is possible that treatment with Cy provided tumor antigen by inducing tumor cell cytolysis. However, due to evidence that DCs do not spontaneously cross-present necrotic cell antigen to CD8 T cells [22], our protocol was designed to include delivery of tumor antigen in the form of tumor cell lysate. In Cy treated mice, the inclusion of lysate mixed with CpG was required to produce the greatest number of anti-AgN2a CD8 T effector cells (Fig. 2b). In other studies, tumor lysate plus CpG was required to produce anti-tumor responses. A murine anti-glioma response occurred when lysate was combined with mouse CpG 2006 or human CpG 7909 [32]. Also, T cell function was inhibited when chemotherapy with CpG-ODN and anti-CD40 were administered without supplemental tumor antigen [21].

One of the goals of this study was to generate a T cell-mediated anti-neuroblastoma response using easily translated immune modulating agents. Cy is a standard of care chemotherapeutic, and in clinical trials, CpG ODNs have been show to be safe when administered as cancer adjuvants [33]. Autologous tumor lysate is difficult to obtain, but it is possible that antigen cross-reactivity provided by allogeneic tumor lysate can substitute as an antigen source. The advantage of using an allogeneic tumor lysate is that it can be prepared as an “off the shelf” agent. In this treatment protocol, treatment with an allogeneic neuroblastoma lysate produced CD8 T cells that were activated in the presence of AgN2a tumor cells (Fig. 5b).

## Conclusions

In summary, the synergistic immune modulating effects of Cy and a lysate/CpG treatment provide T cell-mediated anti-tumor activity against murine neuroblastoma. Both Cy and lysate CpG alter the homeostatic expansion of T cells and modulate DC homeostasis. The synergistic immune modulation provided by Cy with lysate/CpG may boost anti-tumor immunity when combined with standard of care therapy. However, an optimal therapy for neuroblastoma will, most likely, require a combination of chemotherapy and immune activating agents (such as lysate/CpG) in addition to immune therapies that promote anti-tumor T cell memory.

## Methods

### Mice

Male A/J mice, 6–8 weeks of age, were purchased from the Jackson Laboratory. All mice were housed in the Medical College of Wisconsin (MCW) Biomedical Resource Center (Milwaukee, WI), which is accredited by the American Associated for Accreditation of Laboratory Animal Care (AALAC). All animal studies were approved by the Medical College of Wisconsin Institutional Animal Care and Use Committee.

### Tumor cells

An aggressive AgN2a cell line, was produced in our laboratory through sequential *in vivo* passage of Neuro-2a (ATCC) in mice as previously described [33]. To generate established tumors, AgN2a cells were injected subcutaneously (sc) into the hind flank of strain A/J mice. Tumor growth was monitored by standard caliper measurements. Mice were considered moribund and euthanized when tumors reached 250 mm^2^. For some studies, AgN2a cells with stable expression of firefly luciferase (AgN2a FF) were used (pcDNA3.1/firefly luciferase was provided by Dr. Michael Dwinell, Medical College of Wisconsin). The 9464D N-myc overexpressing cell line was developed in the laboratory of Dr. Jon Wigginton at the National Cancer Institute (NCI). The 9464D cell line was derived from a spontaneous tumor that arose in a C57BL/6 N-myc transgenic mouse that was developed by Dr. William A. Weiss (University of California, San Francisco, CA).

### Biophotonic imaging

Mice inoculated with firefly expressing AgN2a cells were ip injected with 50 mg/kg D-luciferin. Luciferase activity was detected using the Xenogen IVIS imaging system (PerkinElmer, Waltham, MA). Regions of interest (ROI) surrounding the tumor bioluminescent signal were drawn using LIVING IMAGE software Version 2.6 (Xenogen). Results are reported as ROI total photon flux.

### Lysate/CpG treatment

Tumor lysate was made from the AgN2a (syngeneic) or 9464D (allogeneic) tumor cell lines. Cells were washed twice with phosphate buffered saline (PBS) and 5 × 10^6^ were suspended in 0.2 ml PBS. For lysis, cells were placed in liquid nitrogen for 60–90 s followed by incubation in a 37 °C water bath for 3–4 min. This freeze/thaw cycle was repeated four times. The lysate was centrifuged at 300 × g for 5 min to pellet cell debris. The cell debris was removed by filtering the lysate through a 0.22 μm filter. The lysate was stored at −80 °C until further use.

Class B CpG-ODN 1826 (5′ TCCATGACGTTCCTGACGTT 3′) was purchased from TriLink Biotechnologies (San Diego, CA). Mice received 50 μg CpG suspended in either 0.2 ml of PBS (CpG only) or in 0.2 ml of tumor cell lysate (lysate/CpG).

### Antibodies and flow cytometry

Cells were stained with combinations of the following monoclonal antibodies: Anti-CD44 (1 M7), anti-IA^k^ (11–5.2) and anti-B220 (RA3-6B2) (BD Biosciences Pharmingen). Anti-CD3 (145-2C11), anti-CD4 (RM4-5), anti-CD8a (53–6.7), anti-CD62L (MEL-14), anti-CD11c (N418), anti-CD11b (M1/70), anti-FoxP3 (FJK-16 s), and anti-Ki67 (eBioscience). 7-aminoactinomycin D (7AAD) was added to each sample as a viability marker. All stained cells were analyzed using a BD LSR II Flow Cytometer System (BD Biosciences, San Diego, CA). The data was analyzed using Flow-Jo software (Tree Star, Inc, San Carlos, CA).

### T cell purification

Spleens or draining lymph nodes (dLN) were isolated, pooled from several mice, and processed into single cell suspensions. Cells were labeled with anti-CD8 conjugated magnetic microbeads (Miltenyi Biotec, Auburn, CA) and were sorted by positive selection using a Miltenyi automated immunomagnetic sorter (autoMACS). The CD8 T cell purity was ~85 %. To increase the purity of CD8 T cells, cells were stained with anti-CD8 antibodies and sorted by flow cytometry using a FACSAria cell sorter (BD Biosciences, San Diego, CA). Sorting by flow cytometry increased the T cell purity to >99 %.

### IFN-γ ELISPOT assay

The numbers of AgN2a-specific IFN-γ producing CD8 T cells were determined by an enzyme-linked immunosorbent spot (ELISPOT) assay (BD Bioscience (San Diego, CA). For this assay, purified T cells were plated in a 96 well plate with 10^5^ live AgN2a target cells. After 24 h, IFN-γ production was quantified using a Cellular Technology Limited (CTL) ImmunoSpot Analyzer (CTL Analyzers, LLC, Cleveland, OH).

### Statistics

ELISPOT and flow cytometry data was analyzed by one-way ANOVA. Survival curves were compared using the log-rank (Mantel-Cox) test. All statistical analyses were calculated using GraphPad Prism 5.0a software (GraphPad, San Diego, CA). P values <0.05 were considered as significant.

## Additional files

Additional file 1: Figure S1. Tumor detection using biophotonic imaging of luciferase expressing tumor cells. Mice were ip injected with the firefly luciferase substrate D-luciferin (50 mg/kg) and imaged for firefly luciferase biophotonic signal using the Xenogen IVIS imaging system. The figure shows serial images of tumor signal in one mouse from each of 3 treatment groups (untreated, Cy only and Cy and lysate/CpG). Additional file 2: Figure S2. Tumor growth is delayed as the Cy dose is increased. Mice were treated as in Fig. 2a. Cy only data from Fig. 3 is graphed. Mice were followed for survival and euthanized when tumor size reached 250 mm^2^. Each graph represents data combined from 2 separate experiments with 5 mice per group. Data was analyzed for statistical significance using the log-rank (Mantel-Cox) test.

## Abbreviations

ODN: Oligodeoxynucleotide
GM-CSF: Granulocyte colony stimulating factor
AgN2a: Aggressive neuroblastoma
HSCT: Hematopoietic stem cell transplant
TLR9: Toll-like receptor 9
Cy: Cyclophosphamide
Lysate/CpG: Tumor cell lysate and CpG
DCs: Dendritic cells
SC: Subcutaneous
IV: Intravenous
FF: Firefly luciferase
dLN: Draining lymph node
IP: Intraperitoneal
EM: Effector memory
