# Supplementary figures and images for: Immune modulating effects of cyclophosphamide and treatment with tumor lysate/CpG synergize to eliminate murine neuroblastoma
Source: J Immunother Cancer. 2015 Jun 16;3:24. doi: 10.1186/s40425-015-0071-3 (PMC4469315; doi:10.1186/s40425-015-0071-3)

Day: 20                      27                      35                      48                      57

Untreated

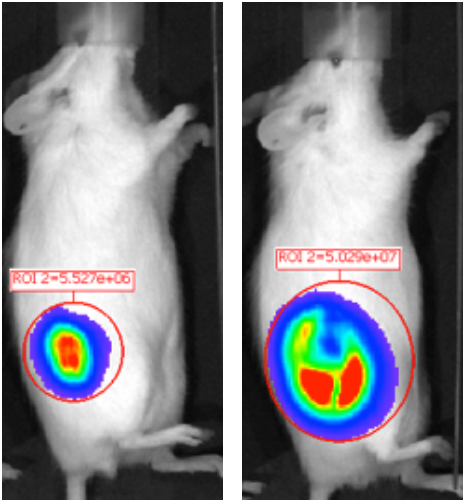

Cy only

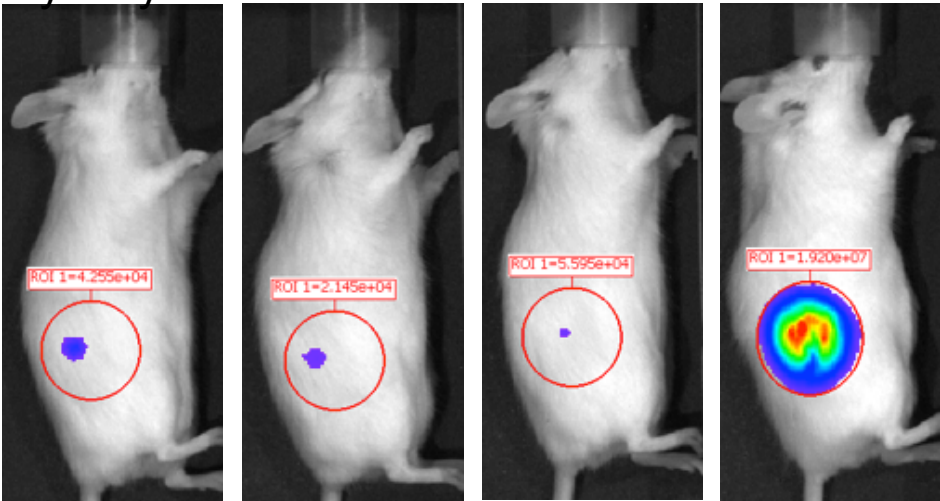

Cy lysate/CpG

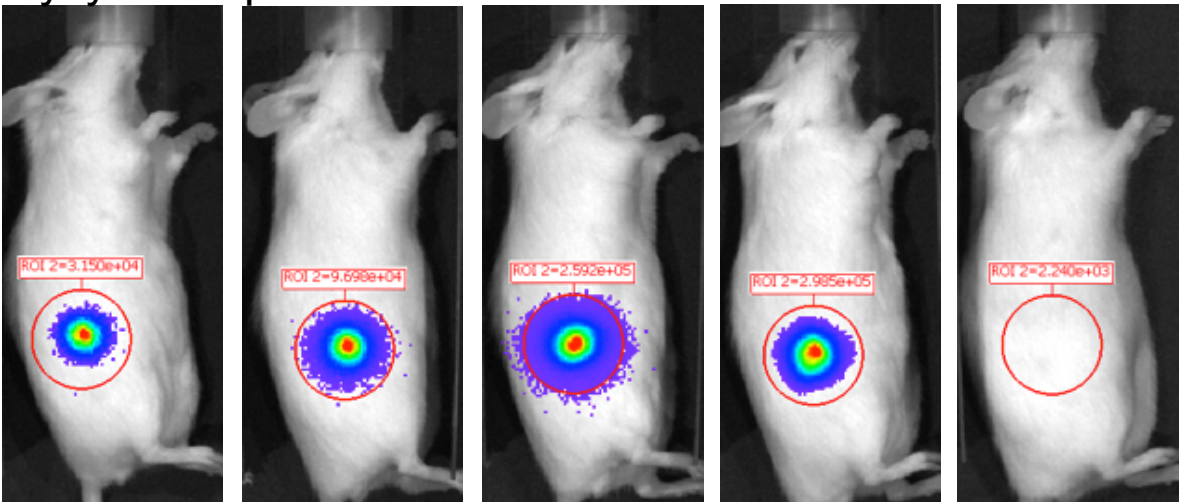

Supplemental Figure 1

Supplement: Additional file 1: Figure S1. — Tumor detection using biophotonic imaging of luciferase expressing tumor cells. Mice were ip injected with the firefly luciferase substrate D-luciferin (50 mg/kg) and imaged for firefly luciferase biophotonic signal using the Xenogen IVIS imaging system. The figure shows serial images of tumor signal in one mouse from each of 3 treatment groups (untreated, Cy only and Cy and lysate/CpG). [file 40425_2015_71_MOESM1_ESM.pdf]

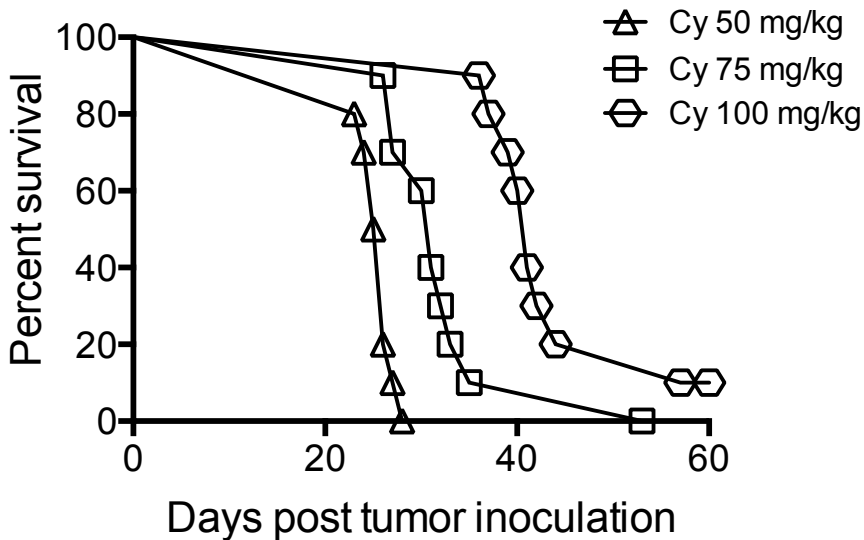

**Supplemental Figure 2**

Supplement: Additional file 2: Figure S2. — Tumor growth is delayed as the Cy dose is increased. Mice were treated as in Fig. 2a. Cy only data from Fig. 3 is graphed. Mice were followed for survival and euthanized when tumor size reached 250 mm2. Each graph represents data combined from 2 separate experiments with 5 mice per group. Data was analyzed for statistical significance using the log-rank (Mantel-Cox) test. [file 40425_2015_71_MOESM2_ESM.pdf]
